# Highly stretchable electroluminescent device based on copper nanowires electrode

**DOI:** 10.1038/s41598-022-13167-4

**Published:** 2022-05-27

**Authors:** Phuong Tran, Nguyen-Hung Tran, Ji-Hoon Lee

**Affiliations:** Division of Electronics Engineering, Future Semiconductor Convergence Technology Research Center, Jeonbuk National University, Jeonju, 54896 Korea

**Keywords:** Nanowires, Electrical and electronic engineering

## Abstract

Although stretchable electroluminescent (EL) devices have been the research hotspots for decades because of their enormous market value in lighting sources and displays, fabrication of the stretchable EL device through a simple, cost-effective, and scalable method still remains an open issue. Here, a novel all solution-processed method is developed to fabricate a high-performance alternative current electroluminescent (ACEL) device based on copper nanowires (Cu NWs). The Cu NW-based electrode exhibited a low resistance change of less than 10% after 1000 stretching cycles at a tensile strain of 30% and the resistance variation of the electrode in one stretching-releasing cycle was less than 1% at the 1000th. To substantiate suitability for the wearable application, the ACEL device was stretched at a tensile strain of 100% and it retained a luminance of 97.6 cd/m^2^. Furthermore, the device works well under different deformations such as bending, folding, rolling, and twisting. To the best of our knowledge, this is the first demonstration of Cu NWs applied in a stretchable ACEL, promising cost-effective electrode materials for various wearable electronics applications.

## Introduction

The development of flexible and stretchable electronic devices in the past decade has stimulated the discovery of new materials and new functions for high-performance wearable devices^1^. The electroluminescent (EL) devices that are deformable by bending, folding, rolling, twisting, or stretching with free form factors are driven by the modern-day demand for new display technology. There are many types of EL devices, including organic light-emitting diodes (OLEDs), polymer light-emitting diodes (PLEDs), perovskite light-emitting devices, and alternative current (AC)-driven EL devices. Compared to other luminescent devices, the AC-driven electroluminescent (ACEL) devices are widely used due to their high contrast, fast response time, and wide viewing angle^2^. Despite the luminance of inorganic phosphor powder EL devices is generally lower than that of OLEDs and PLEDs, it is usually sufficient for most cost-effectiveness lighting and large-area display applications. Moreover, the power consumption and operational lifetime of the ACEL device are the advantages because the charge accumulation can be effectively avoided. Furthermore, in contrast to other light sources, the ACEL devices produce light without generating much heat, which is highly desirable for use in wearable applications.

The ACEL device is operated with an applying alternative electric field across an emissive layer containing phosphor powder dispersed within the dielectric medium, via two parallel electrode plates. Hence, developing stretchable conductive electrode material is critical for EL device fabrication. Over recent decades, various nanomaterials including graphene^3–5^, carbon nanotubes (CNTs)^6–9^, conductive polymer^10^, and metal nanowires (NWs)^11,12^ have been intensively studied for wearable devices, particularly for stretchable display. Among these candidates, metal nanowires are promising materials to achieve the requirements of the conductive material for stretchable devices. In recent years, there has been a rapid rise in the use of Cu nanowires (Cu NWs) for transparent electrodes (TEs) because copper has high electrical conductivity, low cost, and stable global supply^13–16^. In addition, the well-known synthesis and deposition processes of the Cu NWs on the film are not only time-saving compared to ITO sputtering process, but also easy to scale up and cost-effective. However, there have been a small number of studies on copper nanowires (Cu NWs) for stretchable applications. The mechanical stretchability of Cu NW-based electrode has been rarely reported over the past 5 years^17^ and the ACEL fabricated based on Cu NW has not been reported in the literature.

In this report, a stretchable ACEL device, which exhibits high reliability, luminance, and stretchability has been fabricated using the Cu NWs electrodes for the first time. Cu NWs were synthesized via hydrothermal route, and then simply deposited on polydimethylsiloxane (PDMS) substrate by spray coating. The emissive layer of ACEL was prepared and deposited on a stretchable electrode with optimized parameters. The Cu NWs electrode represented superior stretchability under 30% of tensile strain and 1500 stretching cycles. Noteworthy that the resistance was only changed by less than 10% after 1000 cyclic loading. As a result, the ACEL devices showed high luminance of 97.6 cd/m^2^ and stretchability up to 100%. Furthermore, the fabricated EL device can be used under extreme deformation such as bending, folding, rolling, and twisting.

## Results and discussion

**Figure 1:**
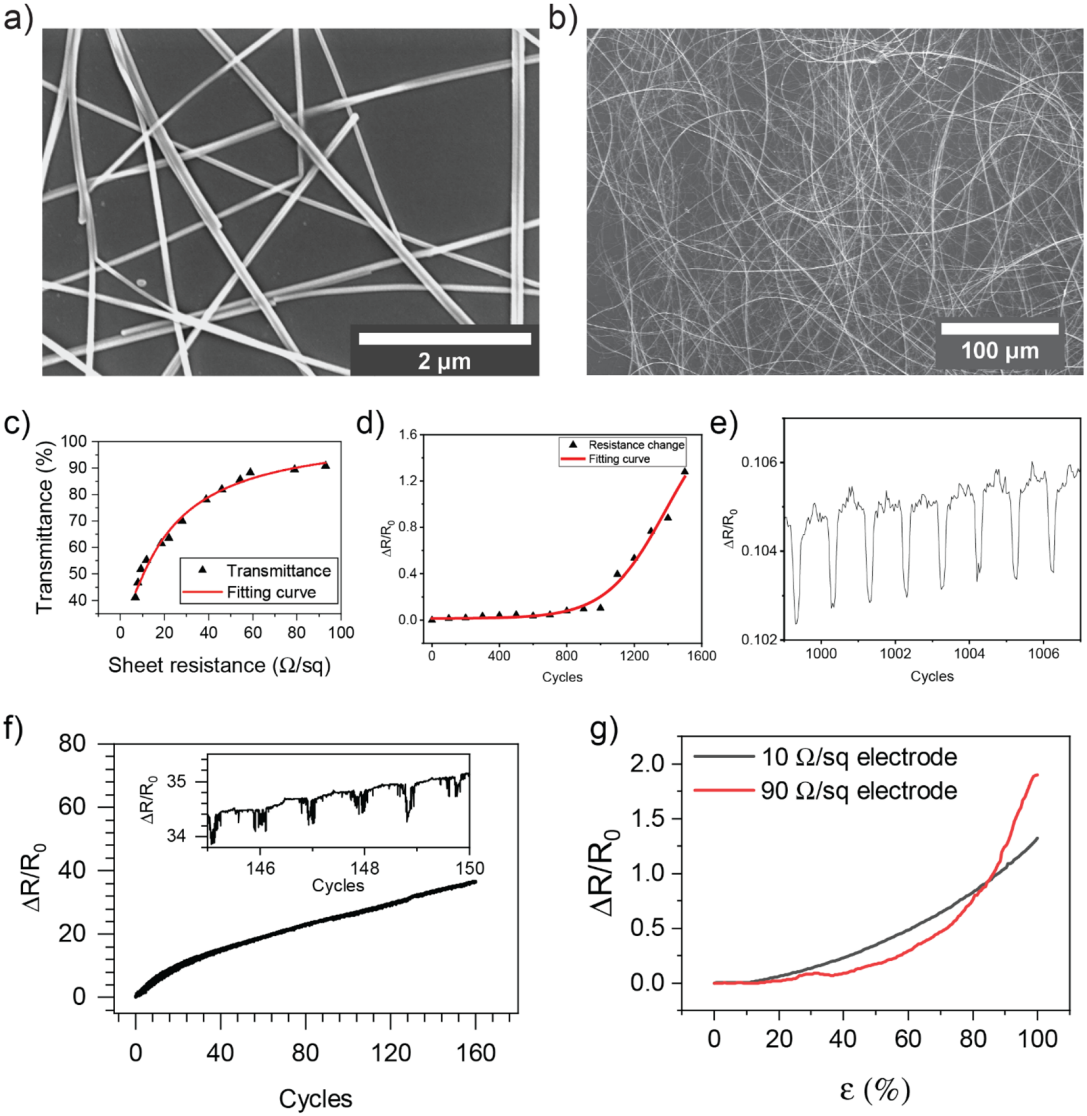
(**a**,**b**) FE-SEM images of synthesized Cu NWs on PDMS substrate with different magnifications. (**c**) Specular transmittance at a wavelength of 550 nm as a function of sheet resistance of the Cu NW electrodes. (**d**) Durability test of the Cu NW-based electrode under the strain of 30%. (**e**) Resistance change of Cu NW-based electrode at 1000th cycle under the strain of 30%. (**f**) Durability test of the Cu NW-based electrode under the strain of 100%, the inset graph shows the resistance change from 145 to 150th cycle (R_0_ = 10 $\Omega$). (**g**) Relative resistance changes under various strains of 10 and 90 $\Omega \backslash \mathrm{sq}$ electrode.

Figure 1a shows the high magnification scanning electron microscopy (SEM) image of Cu NWs coated on PDMS substrate. From the image, the average diameter of synthesized Cu NWs was estimated as 64 nm. From Fig. 1b, it can be revealed that their length was over 200 $\mathrm{\mu m}$ (even some wires were longer than 500 $\mathrm{\mu m}$). This means that our synthesized Cu NWs have an aspect ratio (length/diameter) over 3000—one of the highest aspect ratios of Cu NWs obtained through the HDA-mediated method^18^. In general, the aspect ratio determines the efficiency of the electrode: the longer and thinner of Cu NWs, the greater figure of merit (FoM) of the electrode^19,20^. Besides, Fig. 1b indicates that the NWs were well distributed on the stretchable substrate. In this research, we employed PDMS as the stretchable substrate because of its high transparency, neutral color, large elongation up to 160%, and biocompatibility^21^. However, the PDMS surface has hydrophobic property that may prevent the even spread of the Cu NW ink. Hence, we conducted the plasma treatment on PDMS to form hydroxy functional groups on its surface. The optoelectrical properties of the Cu NW-based stretchable electrode were controlled by changing the spraying time from 1 to 5 s. Figure 1c shows the relationship between the sheet resistance and the transmittance of the electrodes. The sheet resistance varied from 6.9 $\Omega \backslash \mathrm{sq}$ to 90 $\Omega \backslash \mathrm{sq}$, while the specular transmittance of the electrode at 550 nm wavelength was in the range of 41 to 91%, respectively.

To investigate the electromechanical characteristics of the Cu NWs electrode, we observed the resistance change under repeated tensile strain using a motorized stage. Two sides of the electrode which had initial resistance of 10 $\Omega$, were fixed on the motorized stage via acrylic holders and connected to an LCR meter by aluminum tapes and copper wires. The electrode was stretched from 0 to 30% of tensile strain ($\upvarepsilon$) repeatedly at a moving speed of 0.1 mm/s and the resistance was recorded at the interval of 0.5 s. Figure 1d is the plot of the normalized resistance of the electrode after 1500 cycles of stretching. The resistance was increased slowly until the 1000^th^ cycle and then rapidly raised by 1.3 times at the maximum stretching cycles of 1500. It is worthy to note that under 1000 cycles, the resistance was only changed by less than 10%. Figure 1e indicated that the resistance variation of the electrode in one stretching-releasing cycle was less than 1% at the 1000th. Furthermore, the electrode was not lost its conductivity when the strain was increased up to 100%. Figure 1f indicated that the resistance was raised to 360 $\Omega$ after 150 cycles of stretching test at 100% of the strain. It is important to note that the stretchability of the electrode was different from the difference in Cu NW thickness. Two electrodes (10 and 90 $\Omega \backslash \mathrm{sq}$) were stretched to 100% of $\upvarepsilon$ and their resistances were measured (Fig. 1g). When the strain bigger than 80%, the resistance 90 $\Omega \backslash \mathrm{sq}$ sample was increased faster than 10 $\Omega \backslash \mathrm{sq}$ sample and it reached 200% while that of 10 $\Omega \backslash \mathrm{sq}$ sample was 140% at 100% of $\upvarepsilon$. Compared to other research, our Cu NW-based electrode showed better stability under large mechanical deformation^17,20^.

**Figure 2:**
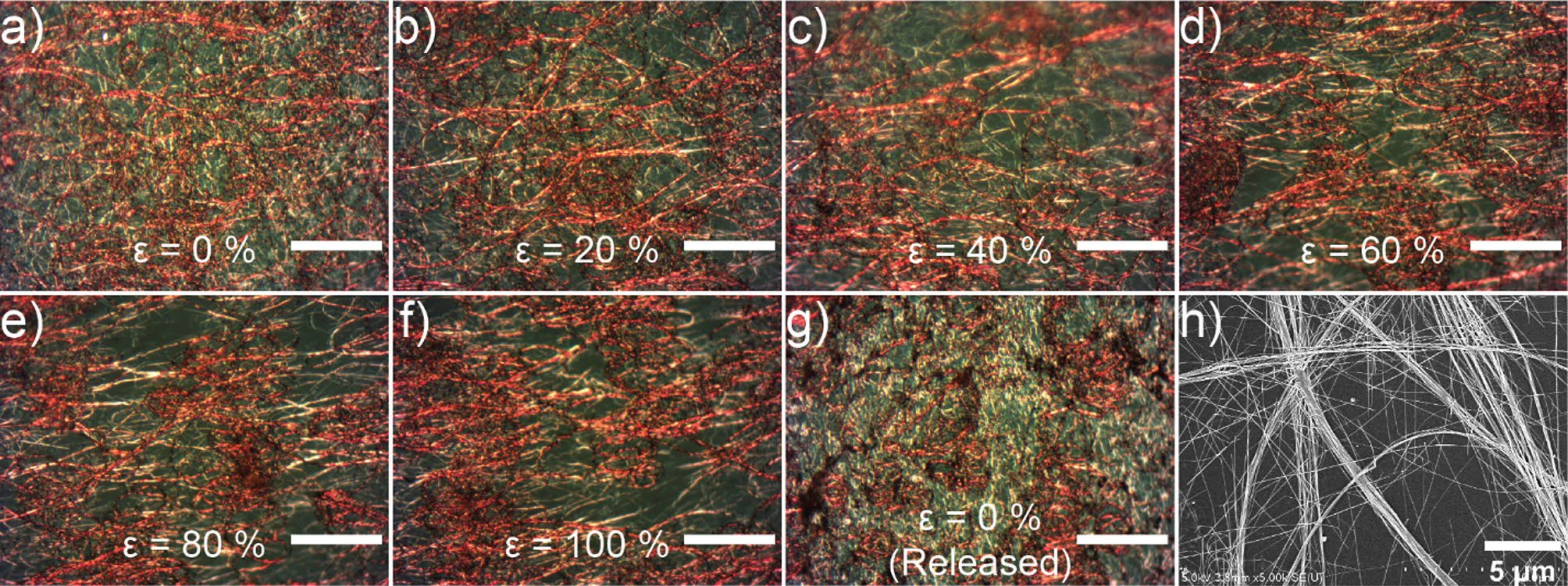
(**a**–**g**) Optical microscopy images of Cu NW electrode under various strains (from 0 to 100%) (Scale bars correspond to 50 μm), (**h**) FE-SEM image of Cu NWs deposited on PDMS by spray coating.

The stability of the Cu NW-based electrode is attributed to the morphology of the Cu NWs after the deposition process by the spray coating method, the reinforcement of the NW network by rope-like NW, and the NWs aspect ratio. To support our claim, we set up the experiment to capture the deformation of the Cu NW network while the electrode is stretching (see Supplementary Information Figure S1). The electrode was fixed on linear stages and stretched from 0 to 100% of ε and the photos were taken by an optical microscope. Figure 2a showed that Cu NWs were rolled in micro curve shapes on PDMS substrate after the spray coating process. The NWs were deformed along the applied tensile strain direction and the distances between rolled NWs were increased when the strain was increased (Fig. 2b–f). After releasing the stress, Cu NWs were recovered to their original formation as shown in Fig. 2g. Figure 2h showed that the NWs were stuck and entwined together after the deposition, thus forming bigger rope-like NWs. When the applied strain was bigger than 60% (Fig. 2e,f), single NWs were observed to break, while these rope-like NWs were maintained and allowed the flow of charges through. The rope-like NWs reinforced the NW network and protected the electrode from losing conductivity. In addition, as mentioned above, the aspect ratio of synthesized Cu NW is over 3000, and this high aspect ratio is well known to support the mechanical stability of the electrode^22^.

**Figure 3:**
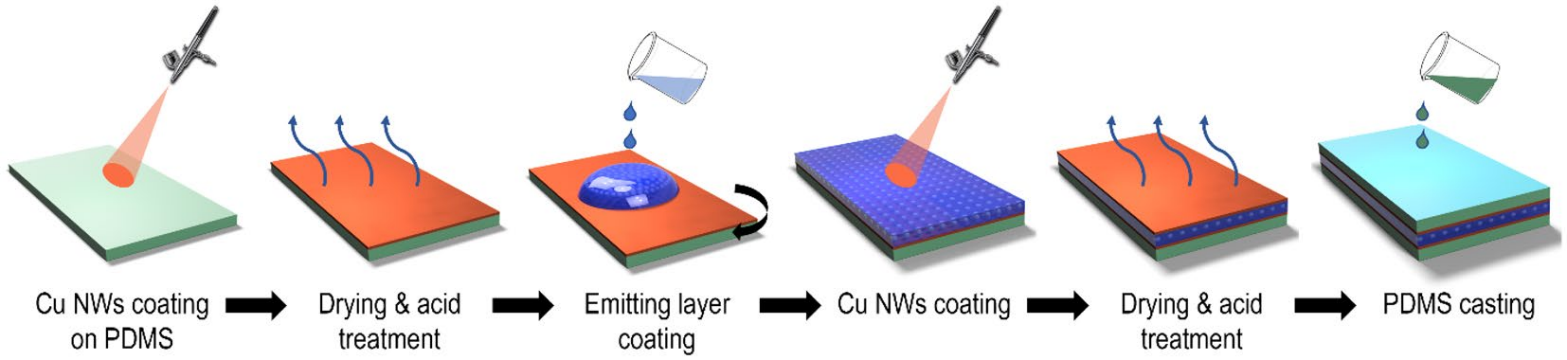
Schematic of the electroluminescent device fabrication process.

**Figure 4:**
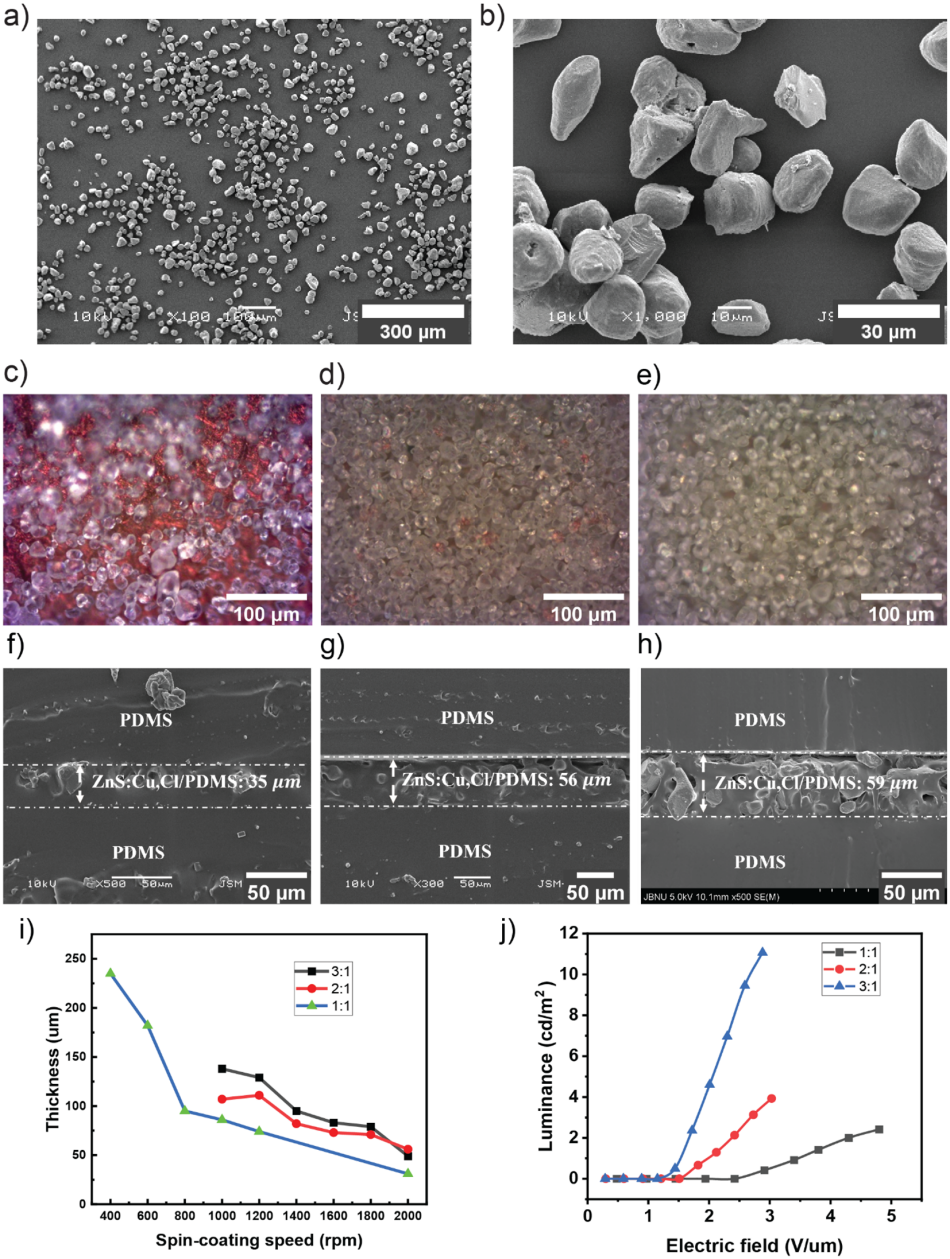
(**a**,**b**) FE-SEM of ZnS:Cu,Cl particles with different magnifications. (**c**–**e**) Optical microscopy images of ZnS:Cu,Cl particles coated on Cu NW-based electrode; the weight ratio of ZnS:Cu,Cl/PDMS was 1:1, 2:1, and 3:1 in (**c**–**e**), respectively. (**f**–**h**) cross-sectional SEM images of fabricated EL devices with the corresponding weight ratio of ZnS:Cu,Cl/PDMS. (**i**) Thickness of emissive layer prepared with the different weight ratios of ZnS:Cu,Cl/PDMS and spin-coating speed. (**j**) The luminance of EL devices prepared with the different weight ratios of ZnS:Cu,Cl/PDMS.

To evaluate the feasibility of the fabricated electrodes, the Cu NW-based electrodes were tested for stretchable electroluminescent (EL) devices. ACEL devices were prepared via an all-solution process (as shown in Fig. 3). Here, we used well-known phosphor ZnS:Cu,Cl particles as an emission layer. ZnS:Cu,Cl particles were dispersed in PDMS, spun on Cu NWs electrode, and cured to form the emissive layer. The average dimension of the particles was 25 $\mathrm{\mu m}$ from FE-SEM images (Fig. 4a,b). We mixed ZnS:Cu,Cl particles in PDMS in the different weight ratios of 1:1, 2:1, and 3:1. The mixtures were placed in a vacuum chamber for 30 min to eliminate air bubbles from the gel. Figure 4c–e are the optical microscopy images of the emissive composite coated on Cu NW electrode after spin-coating at 2000 rpm. The distribution of the particles was uniform, and the concentration of the particles was increased from Fig. 4c–e. Moreover, no air bubble was observed in the images. The emissive layer was homogeneous and the particles were well adhered to the PDMS matrix. Figure 4f–h show the cross-sectional SEM images of the EL devices fabricated with the weight ratio of ZnS:Cu/PDMS from 1:1 to 3:1. The thickness of the emissive layers varied from 35 to 59 $\mathrm{\mu m}$. Increasing the weight ratio of ZnS:Cu/PDMS would increase the viscosity of the mixture, resulting in thicker emissive layer after spin-coating.

The thickness of emissive layers fabricated with the different weight ratios of ZnS:Cu/PDMS was plotted vs. spin-coating speeds (Fig. 4i). As shown in Fig. 4j, an increase of the weight ratio of ZnS:Cu led to a significant enhancement in the luminance and the maximum luminance was achieved at the weight ratio of 3:1. When the weight ratio was bigger than 3:1, the viscosity of the mixture largely increased, making it difficult for device fabrication. Furthermore, under the high weight ratio of phosphor, the ZnS:Cu,Cl particles could not be sufficiently insulated with PDMS. This is analogous to an increased ZnS:Cu,Cl particle size in the polymer matrix^23^. Therefore, the weight ratio of 3:1 and the spin-coating speed of 2000 rpm were kept as optimized parameters to deposit the emissive layer on Cu NW electrode.

**Figure 5:**
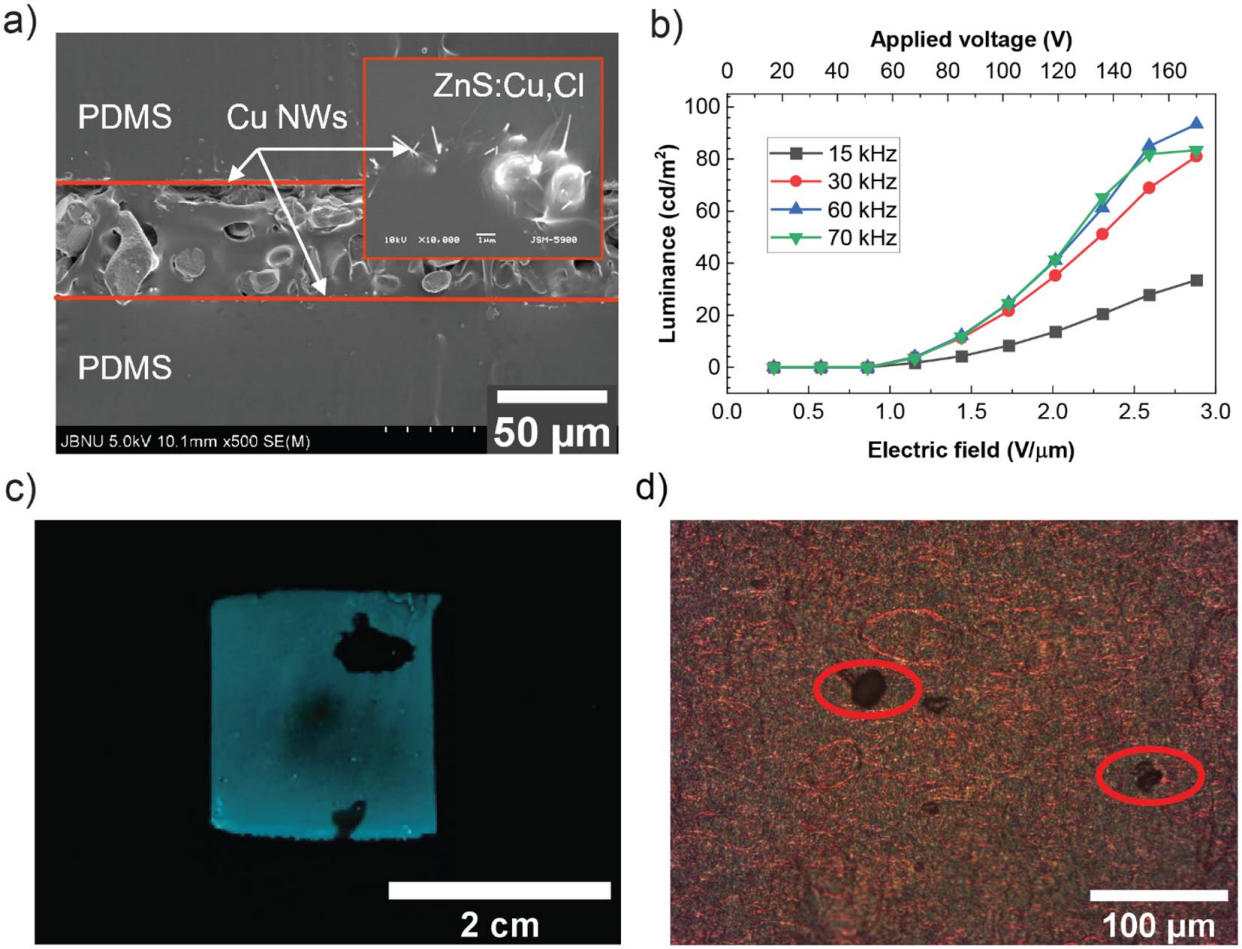
(**a**) Cross-sectional SEM image of the fabricated ACEL device with the weight ratio of ZnS:Cu/PDMS = 3:1; magnified image is presented in the inset. (**b**) Luminance measured with increasing bias voltage under various frequency. (**c**) Optical image of the emitting sample at 4 V/$\mathrm{\mu m}$ of electric field. (**d**) Microscopy image (top view) of the emitting sample at 4 V/μm of electric field.

After a thorough study to optimize the device architecture, we fabricated a device in which the weight ratio of ZnS:Cu/PDMS was 3:1 and the thickness of the emissive layer was 59 $\mathrm{\mu m}$. A cross-sectional SEM image of the optimized device is shown in Fig. 5a. The ZnS:Cu,Cl particles were uniformly dispersed in the cured PDMS matrix and sandwiched between a pair of Cu NWs/PDMS films. The PDMS layer penetrates through the Cu NW network, resulting in a stable Cu NW electrode that is fully embedded in the PDMS (inset image of Fig. 5a) and adheres well to the bottom PDMS substrate. Strong and stable bonding between each layer could help maintain optimum device performances during different mechanical deformations such as stretching, bending, twisting, and folding^23^. In the fabricated device, the resistance of the bottom electrode was low as 10 $\Omega /\mathrm{sq}$, while a 60 $\Omega /\mathrm{sq}$ electrode was used as the top electrode. The top electrode exhibited 86.7% of transmittance and more light was emitted through the top electrode.

Figure 5b shows the luminance vs. the applied electric field. The luminance was monotonically increased with a stronger electric field, which means that an electrically stable ACEL device was successfully fabricated via our approach. The dependence of the luminance (*L*) on the applied voltage (*V*) is expressed by the relation:

$$L={L}_{0}\,{exp}\left(-\frac{\beta }{{V}^\frac{1}{2}}\right)$$

where ${L}_{0}$ and $\beta$ are constants determined by the device^24^. The value of these constants depends on the particles size of the phosphors, the concentration of the EL powder in the dielectric, the dielectric constant of the matrix material, and the device thickness. The experimental data fit well with the expression, suggesting a rapid increase of emission at high drive voltages. The device started emitting light at a bias voltage of 68 V, and the emission luminance increased dramatically, reached to a maximum value of 170 V. Notice that dielectric breakdown may occur under excessively high voltage, which poses a practical limit on the luminous intensity. Our experimental data provided that when the applied electric field was greater than 4 V/$\mathrm{\mu m}$, the sparks were observed. Figure 5c shows the device which had a dark zone appeared after working at an electric field of 4 V/$\mathrm{\mu m}$ for 2 min. Besides the large area destroyed by the electric breakdown, the micro dark spots were shown under the microscope (Fig. 5d). The emission characteristics of the ACEL device were also directly influenced by the frequency of the alternative current. At the applied electric field of 3.5 V/µm, the luminance was increased from 30 to 97.6 cd/m^2^ corresponding to the increase of the frequency from 15 to 60 kHz (Fig. 5b).

**Figure 6:**
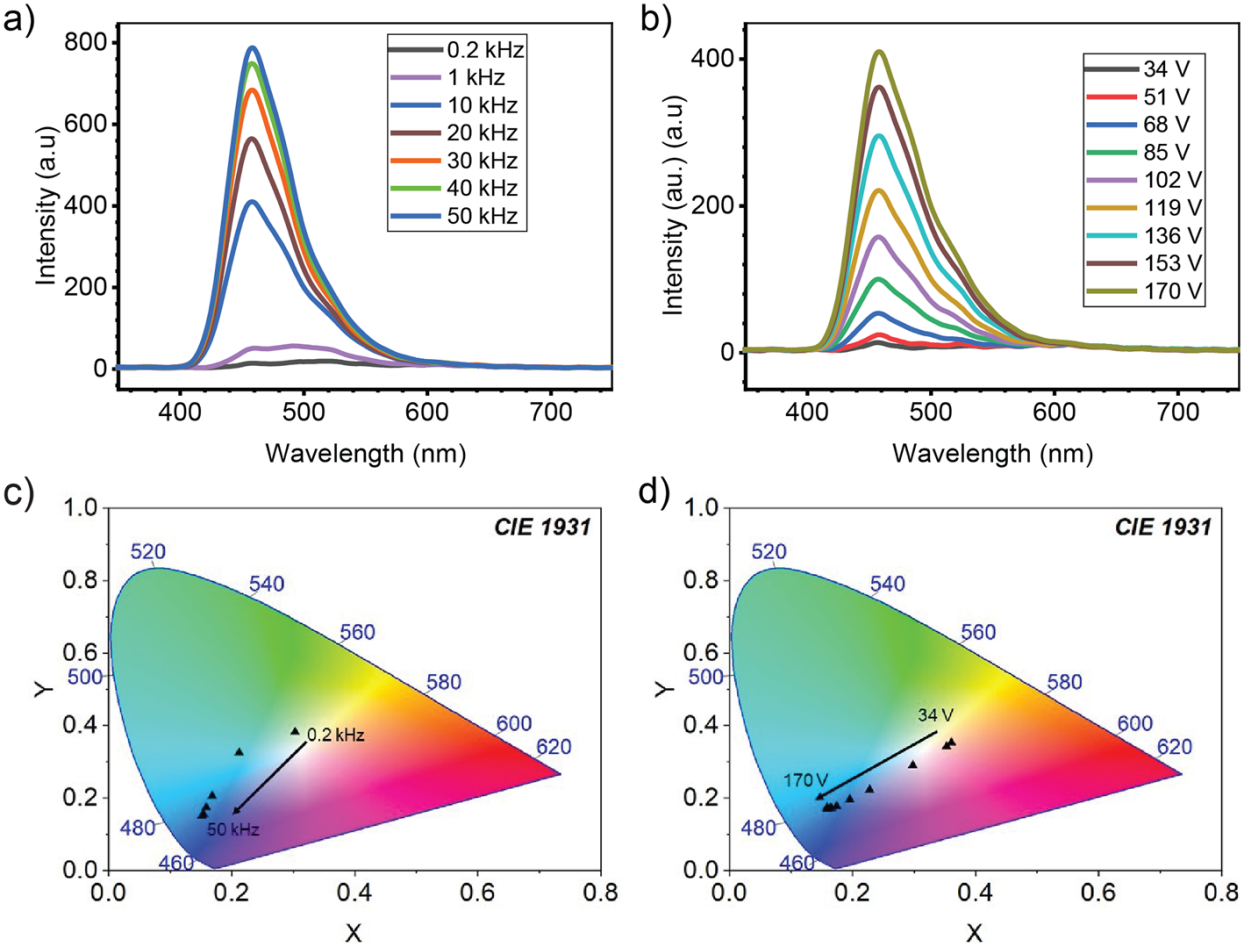
Emission spectrums of the EL devices as functions of (**a**) applied frequency, (**b**) applied voltage. CIE 1931 color space chromaticity diagrams of EL devices by changing (**c**) applied frequency, (**d**) applied voltage.

We need to note that the emitting color spectrum of the EL devices changes with frequencies. In Fig. 6a, the spectrums of the EL devices shows the shift of the intensity peak position. For better viewing of the change, we transformed the emission spectrums into a color space chromaticity diagram as determined by the Commission Internationale d’Éclairange (CIE) in 1931 (Fig. 6c). The light is green–blue at low frequencies and shifts to blue at high frequencies. Under an electric field excitation, it is understood that the charge carrier is accelerated with high energy. This results in the blue-shift of the color with an increasing frequency because the carrier movement at the conduction band and trap level of ZnS is frequency-dependent^24^. A similar color shifting was also found by changing the applied voltage (Fig. 6b,d).

**Figure 7:**
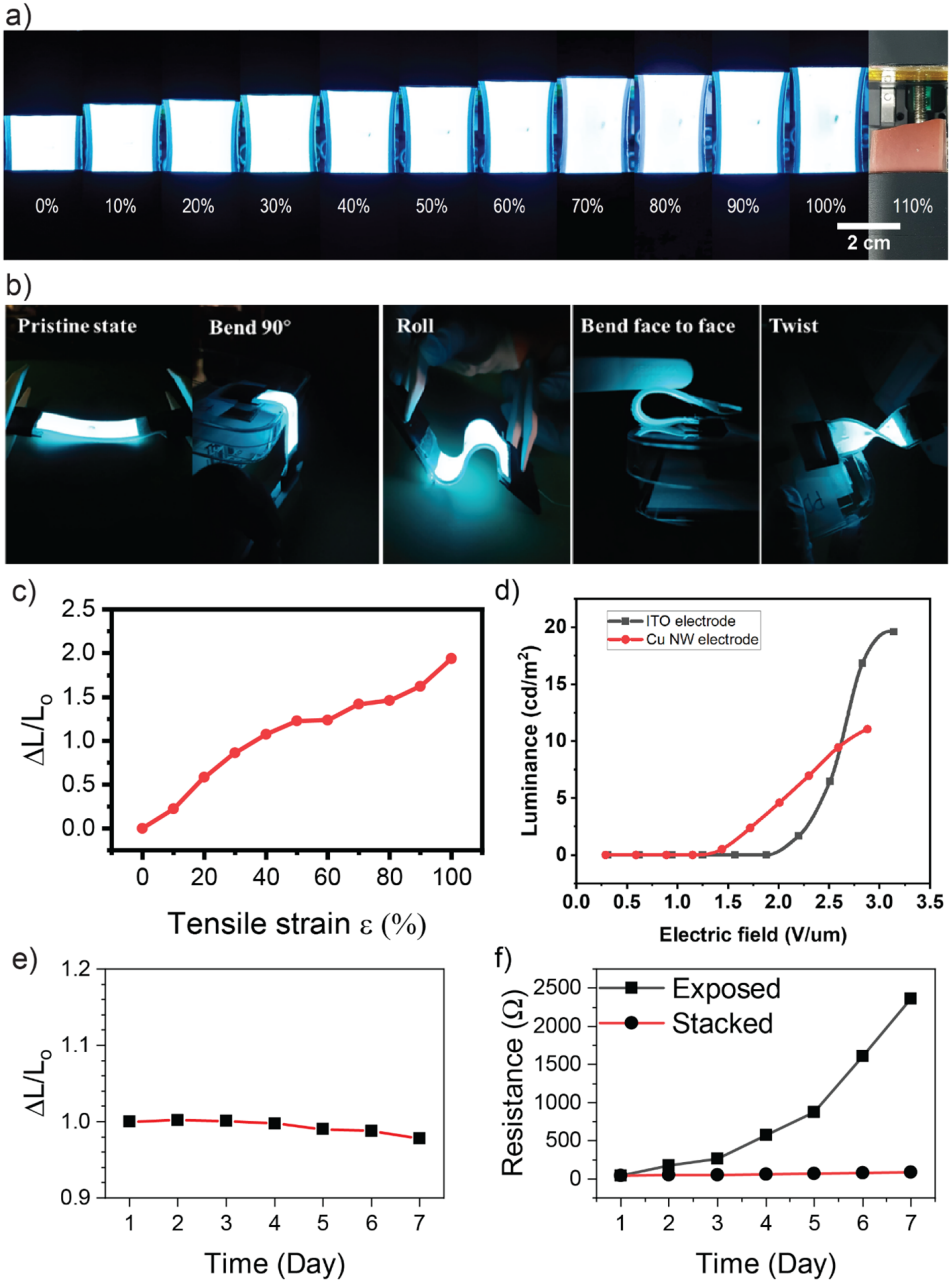
(**a**) Photographs of a light-emitting device under various strain values. (**b**) Snapshot of the light-emitting device under different deformations. (**c**) Luminance changes with the strain applied up to 100%. (**d**) Luminance of Cu NW-based electrode EL device compared to ITO-based electrode EL device. (**e**) Luminance changes of the EL device placed in room temperature, ambient conditions for 7 days. (**f**) The resistance changes of Cu NW electrode exposed directly in the air and Cu NW electrode stacked in the EL device for 7 days.

As the main goal of this paper, the performance of the EL device was evaluated under large-strain deformation (Fig. 7). The sample was turned on and the luminance was simultaneously measured at various tensile strains. Similar to the stretching test of the Cu NW-based electrode, the EL device was attached to a motorized stage via two acrylic holders. The AC power supply was connected to the device through copper wires and aluminum conductive tapes. The device was stretched up to 110% at a constant speed of 0.1 mm/s and its luminance was measured as shown in Fig. 7a,c. The devices showed perfectly stretching without exhibiting any critical damages or degradation of emission, even at the strain value of 100% Fig. 7a. The emission intensity continuously increased with increasing strain mostly due to the decrease of the device thickness Fig. 7c, which coincides with Poisson’s effect. When the EL device is stretched in-plane, its dimension along the stretching direction would increase, however, its thickness which is perpendicular to the stretching direction would be reduced to $\left(1-\vartheta \varepsilon \right){t}_{0}$; where $\vartheta$ is Poisson’s contraction ratio, $\varepsilon$ is applied strain, and ${t}_{0}$ is original device thickness.

Beside stretching, the fabricated EL device is deformable by bending, folding, rolling, and twisting as illustrated in Fig. 7b. Compared to other research, our fabricated EL device showed high stretchability and luminance. For example, Ag NWs-MXene-based EL device fabricated by Park et.al showed a luminance of 79 cd/m^2^^25^; another work from Zang et.al reported their Fish gelatin-based EL device with a luminance of 56 cd/m^2^^26^. Stretchable ACEL devices fabricated using AgNW/CF coatings on PDMS reported by Chen et.al exhibited the stretchability up to 60%^27^ while the device fabricated using AgPdCu thin film showed the stretchability of only 40%^28^. In addition, we compared our EL device to another device fabricated based on a commercial flexible ITO film and plot the luminance in Fig. 7d. It is shown that the EL based on Cu NWs could start emitting light at a lower electric field than the EL device based on commercial ITO because the Cu NWs electrodes have significantly lower sheet resistance than that of ITO film (10–60 $\Omega /\mathrm{sq}$ compared to 472 $\Omega /\mathrm{sq}$). The stability of the device was examined by placing the EL device at room temperature, ambient conditions and measuring the luminance for 7 days. Figure 7e shows that the luminance of the device decreased by 3% compared to the initial luminance (L_0_ = 6.77 cd/m^2^). The degradation of the device may be caused by the attenuation of ZnS:Cu/Cl particles^29^ and the resistance change of stretchable electrode. The oxidation of Cu NWs has been reported as a critical issue^17^. In Fig. 7f, the resistance of Cu NW electrode exposed directly to the air at room temperature, ambient conditions was rapidly raised to over 2 $\mathrm{k\Omega }$ after 7 days. Interestingly, we found that the resistance of the electrode stacked in the device was also increased, but the growth rate was significantly smaller than that of the exposed electrode. The Cu NW network in the EL device was sandwiched between the PDMS substrate and the ZnS:Cu/Cl emission layer, which can protect the electrode from oxygen and moisture. Hence, the device electrodes were maintained its resistance as low as 90 $\Omega$ after one week. From the above results, it can be concluded that the proposed EL device is reliable and stable while working with large deformation (up to 100% of the tensile strain) and it is possible to be used as a wearable display device. To the best of our knowledge, this is the first demonstration of ACEL device using Cu NWs in the literature.

In conclusion, we presented an all-solution-processed method for fabricating a high-performance ACEL device using Cu NW-based electrode. The Cu NWs showed morphology of 64 nm in diameter and more than 200 μm in length. The sheet resistance varied from 6.9 to 90 $\Omega /\mathrm{sq}$ while the specular transmittance of the electrode at the wavelength of 550 nm was in the range of 41 to 91%, respectively. Additionally, the NW network resulted in stable conductivity at ε up to 30% and long-term durability (1000 cycles) of the electrode. A luminance of 97.6 cd/m^2^ and stretchability up to 100% was achieved from the ACEL device. Furthermore, the device retained the emission well under different deformations such as bending, folding, rolling, and twisting.

## Method

### Material

D-( +)-glucose (#G8270, C_6_H_12_O_6_, 99.5%), n-hexane (#296,090, anhydrous, 95%), were procured from Sigma-Aldrich. Copper (II) chloride dihydrate (#18605S0301, CuCl_2_·2H_2_O), acetic acid (1002-4400), isopropyl alcohol (#5035-4404, IPA, 99.5%) was purchased from Daejung Chemical & Metal (South Korea). 1-hexadecylamine (#B22459, HDA 90%) was purchased from Alfa Aesar. Silicone Elastomer Kit (31-00858-01, DOW CORNING) was purchased from 4Science (South Korea). ZnS:Cu,Cl particles were purchased from Lonco.

### Cu NW-based stretchable electrode fabrication

Typically, Cu NWs were synthesized by the hydrothermal method^30^. First, 0.42 g of copper (II) chloride dihydrate was dissolved in 200 mL DI water prepared in an Erlenmeyer flask, then 5.2 g of HDA were subsequently added to the flask and stirred at 500 rpm at room temperature (25 °C). After 24 h of stirring, the mixed solution was changed to a sky-blue color. Next, 1 g glucose was added into the solution and kept stirring for 3 min. After that, the flask was sealed by a screwcap and placed in an oven at 100 °C for 7 h 20 min. The final solution was changed to reddish brown and further cleaned by DI water, n-Hexane, IPA for 3 times^31^ by centrifugation for 5 min at 2500 rpm.

The silicone elastomer was prepared by mixing the elastomer base and curing agent with a weight ratio of 10:1. After stirring for 10 min, the gel was degassed for 30 min and casted on a soda-lime glass which was cleaned subsequently in acetone, IPA, DI water using an ultrasonicate cleaner. Then the PDMS on glass was placed in the oven at 35 °C for 10 h. Before spraying Cu NWs on PDMS, the PDMS substrates were treated by oxygen plasma.

The Cu NW ink with a concentration of 1 mg/ml was obtained by dispersing the cleaned Cu NWs in IPA. Then Cu NWs were sprayed with a 0.4 mm nozzle 10 cm away from PDMS substrate at a constant pressure of 0.2 atm. The Cu NW sprayed sample was dried in the atmosphere for 30 s. In order to make the nanowire network conductive, the Cu NW-coated PDMS film was dipped into glacial acetic acid for 5 s and dried by a hot-air gun (Bosch GHG500 was set at 90 °C) in a few seconds.

### Electroluminescent device fabrication

The device fabrication was illustrated in Fig. 3. The emitting layer was made by mixing the ZnS:Cu,Cl powder with the PDMS liquid in weight ratios of 1:1, 2:1, 3: 1. Then the gel was spun on the as-fabricated Cu NW-based electrode at 400–2000 rpm for 40 s. Next, the emitting layer was cured in the oven at 35 °C for 10 h. Cu NW ink was directly spin-coated on the light-emitting layer to form the top electrode followed by the same with the bottom electrode fabrication procedure.

### Characterization

The morphology of the electrode was investigated by field emission scanning electron microscope (FE-SEM) (SU-70, HITACHI). The emission spectrums were obtained in the range of 350 ~ 750 nm of wavelength by a UV–visible spectrometer (SV2100R-KMAC) (see Supplementary Information Figure S2). The optical specular transmittance of the Cu NW-based electrodes was measured using a UV/vis spectrophotometer (UV-2600, SHIMADZU). The sheet resistance of the FTEs was measured via 4-point probe method (CMT-SR2000N, AiT Co.,Ltd), and the resistance of the electrode was measured by a multimeter and LCR meter (ZSM2376, NF). The luminance of the device was measured by a luminance meter (TES-137).

## Supplementary Information

Supplementary Information.
