# Supplementary material for: Highly stretchable electroluminescent device based on copper nanowires electrode
Source: Sci Rep. 2022 May 27;12:8967. doi: 10.1038/s41598-022-13167-4 (PMC9142487; doi:10.1038/s41598-022-13167-4)
Supplement: Supplementary file 1 — Supplementary Information. [file 41598_2022_13167_MOESM1_ESM.doc]

Supplementary Information

Highly stretchable electroluminescent device based on copper nanowires electrode

Phuong Tran, Nguyen-Hung Tran*, and Ji-Hoon Lee*

Future Semiconductor Convergence Technology Research Center, Division of Electronics Engineering, Jeonbuk National University, Jeonju 54896, Korea

**
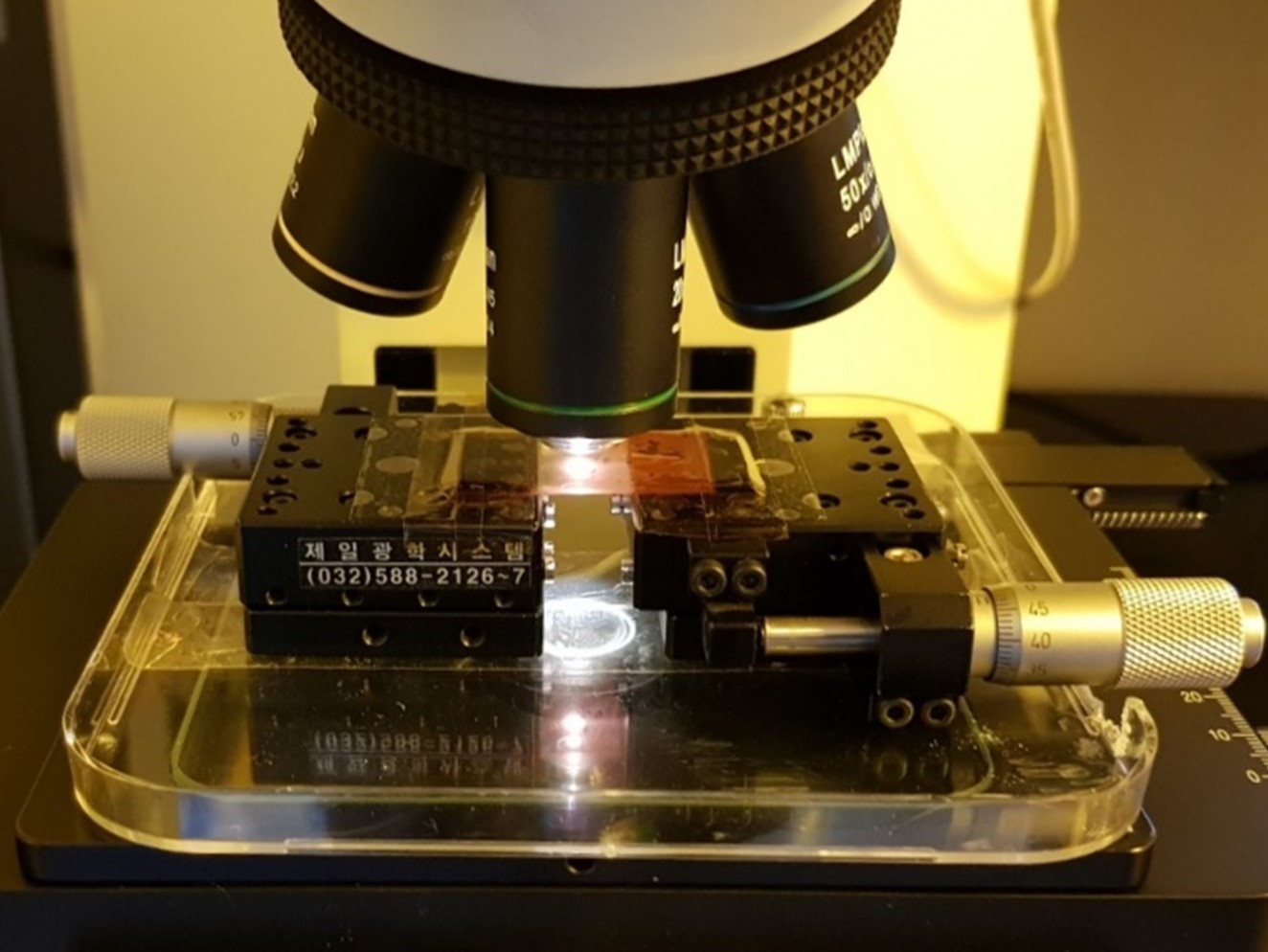
**

**Figure S1**. Experimental setup for capturing Cu NWs network while the electrode is stretching.


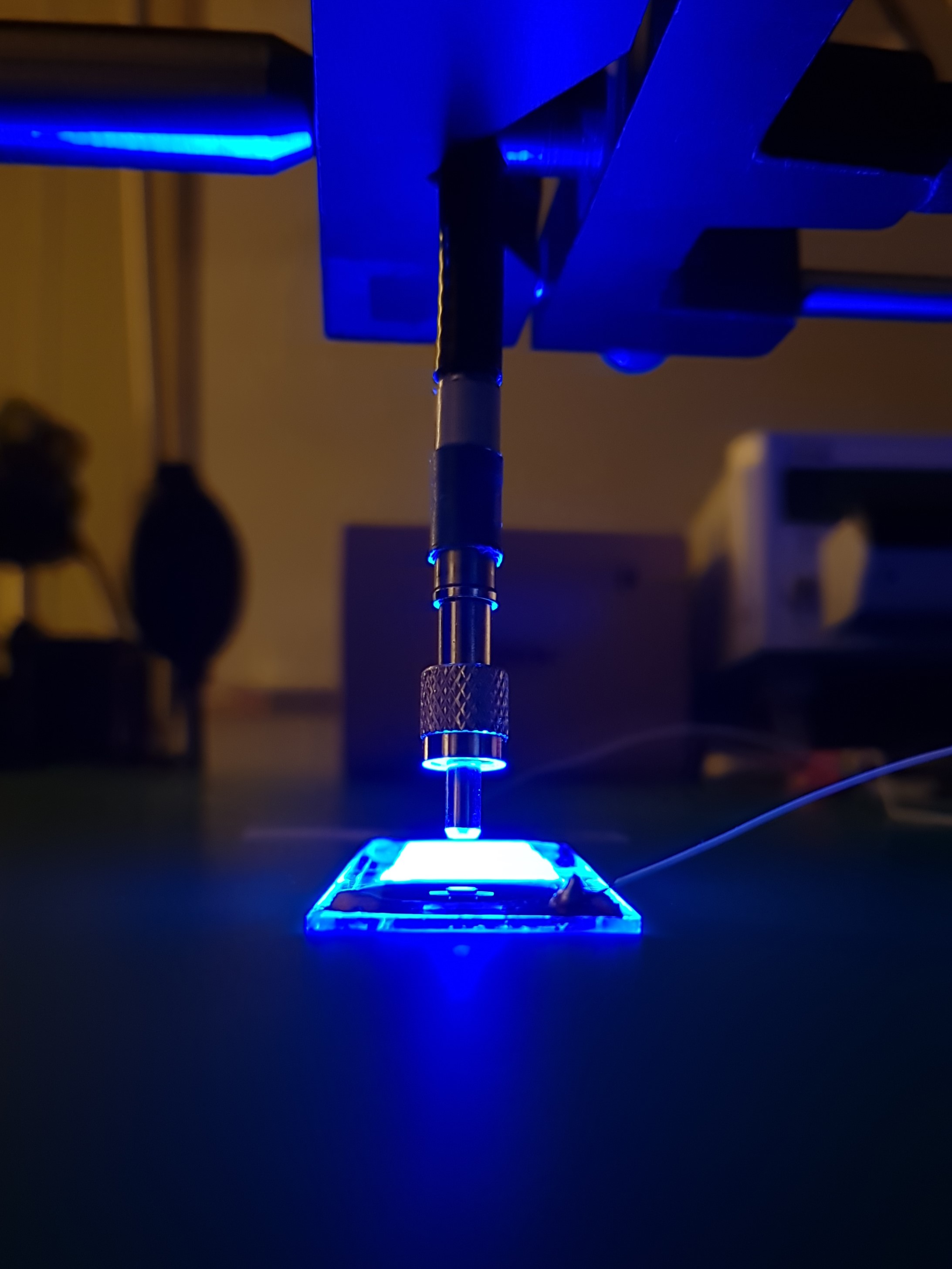


**Figure S2**. Experimental setup of emission spectrums measurement of the EL devices.
